# LncRNA UNC5C-AS1 inhibits angiogenesis and induces endothelial apoptosis via the miR-148a-3p/EMP1 axis in preeclampsia

**DOI:** 10.1080/19336918.2026.2622820

**Published:** 2026-02-06

**Authors:** Yang Wang, Yan Gao, Lingfang Liu, Ke Ma, Yingying He, Hongbo Qi, Xuemei Zhang

**Affiliations:** a Department of Obstetrics, The First Affiliated Hospital of Chongqing Medical University, Chongqing, P. R. China; b Department of Obstetrics and Gynecology, Chongqing Key Laboratory of Maternal and Fetal Medicine/Joint International Research Laboratory of Reproduction & Development, Ministry of Education/The Innovation and Talent Recruitment Base of Maternal-Fetal Medicine, The First Affiliated Hospital of Chongqing Medical University, Chongqing, P. R. China; c Department of Obstetrics, Sichuan Provincial Women’s and Children’s Hospital, The Affiliated Women’s and Children’s Hospital of Chengdu Medical College, Chengdu, P. R. China

**Keywords:** EMP1, HUVEC, lncRNA UNC5C-AS1, miR-148a-3p, preeclampsia

## Abstract

Preeclampsia (PE) is a severe pregnancy complication with unclear molecular mechanisms. Our research investigated the effect of UNC5C-AS1 on human umbilical vein endothelial cell (HUVEC) function in PE. UNC5C-AS1 was downregulated in PE placentas. Upregulating UNC5C-AS1 promoted HUVEC migration, invasion, tube formation, and the expression of vascular permeability factors, while UNC5C-AS1 silencing exhibited an opposite effect. UNC5C-AS1 directly targeted the miR148a3p/EMP1 axis. MiR-148a-3p was up-regulated and EMP1 was downregulated in PE. The regulatory effects of UNC5C-AS1 overexpression on HUVEC functions were reversed by miR-148a-3p mimics, and this reversal was subsequently rescued by EMP1 upregulation. UNC5C-AS1 overexpression ameliorated tissue damage in the PE mouse model. UNC5C-AS1 alleviated the PE-associated injury and modulated HUVEC function by targeting miR-148a-3p/EMP1 axis.

## Introduction

Preeclampsia (PE), a pregnancy disorder developing after 20 weeks of gestation, is defined by new-onset hypertension with proteinuria [1]. The prevalence of PE has risen to approximately 2–8% and represents a major contributor to adverse maternal and perinatal outcomes [2]. Prior reports have evidenced that PE pathogenesis involves multiple factors, including abnormal remodeling of spiral arteries, systemic inflammation, endothelial dysfunction and excessive trophoblast apoptosis [3,4]. Unfortunately, the exact pathogenesis of PE has not been fully elucidated. Therefore, in-depth explorations into the pathogenesis of PE are of great significance for searching effective therapeutic targets.

Long noncoding RNAs (lncRNAs) are a class of RNA transcripts with >200 nucleotides that lack the ability to code for proteins involving in multiple pathological processes via epigenetic regulation [5]. Accumulating evidence demonstrates that lncRNAs can act as competitive endogenous RNAs (ceRNAs), which function as molecular sponges to sequester microRNAs (miRNAs), thereby modulating the post-transcriptional repression of miRNA target genes. For instance, Zhai et al suggested that lnc RNA NR2F1-AS1 promoted breast cancer metastasis via regulating miR-25-3p/ZEB2 axis [6]. Besides, emerging evidence reveals that lncRNAs contribute to PE, and several lncRNAs exhibit differential expression. A previous study found that lncRNA WT1-AS influences trophoblast behavior by targeting miRNA-186-5p/CADM2 axis [7]. Research from Chen et al. suggested that lncRNA PAXIP-AS1 promotes the invasive and migratory capacity of trophoblast cells through regulation of miR-210-3p/BDNF pathway [8]. In this investigation, we identified a newly differentially expressed lncRNA, lncRNA UNC5C-AS1 (UNC5C-AS1, also known as lnc-PDHA2), in PE by lncRNA microarray analysis. However, the precise role of UNC5C-AS1 in PE pathogenesis requires further investigation.

MicroRNAs (miRNAs) are highly conserved single-stranded RNA that can lead to translational repression or degradation by binding to target mRNAs [9]. As key regulators of cellular processes such as differentiation, apoptosis, and immune responses [10], miRNAs have been widely studied in PE. Studies have identified dysregulated miRNAs in the placental tissues of PE patients, including miR-372-3p [11], miR-21 [12] and miR-296-3p [13]. By overexpressing UNC5C-AS1 in cells and performing transcriptome sequencing, we identified miR-148a-3p as a differentially expressed miRNA. Bioinformatics analysis further predicted miR-148a-3p as a candidate miRNA with potential binding sites on UNC5C-AS1. MiR-148a-3p, belonging to the miR-148/152 family, regulates endothelial cell function through modulation of lipid metabolism and contributes to tumor migration and invasion [14]. For example, miR-148a-3p was reported to promote ferroptosis in colorectal cancer cell via targeting SLC7A11 [15]. Nevertheless, the potential mechanism of miR-148a-3p in PE has not been reported.

Epithelial membrane protein 1 (EMP1) belongs to the family of epithelial membrane proteins (EMPs), which are considered to be cellular junction proteins at the plasma membrane [16]. Recently, research has found that EMP1 plays a broad regulatory role in cell biological functions. For instance, Wang et al. discovered that silencing ETV4 reduces the transcriptional activation of EMP1, thereby suppressing the PI3K/AKT/mTOR signaling cascade and facilitating autophagy and apoptosis [17]. A study from Liu et al. revealed that EMP1 deficiency enhances the metastasis of human bladder cancer cells through activating PPAR gamma signaling pathway [18]. In addition, starBase was used to predict miR-148a-3p target genes, and EMP1 was identified as a candidate differentially expressed in PE. Nevertheless, the potential relationship between miR-148a-3p and EMP1 in modulating cellular migration and invasion during PE pathogenesis has yet to be elucidated.

Therefore, we hypothesized that UNC5C-AS1 might regulate HUVEC migration, invasion, and tube formation through regulating miR-148a-3p/EMP1 axis in PE progression. This study clarified the function of UNC5C-AS1 in a PE cell model and PE mice, as well as explored the interaction of UNC5C-AS1 with miR-148a-3p/EMP1 in PE.

## Materials and methods

### Clinical samples

A total of six pairs of placental tissue samples were obtained from patients with PE (*n* = 6) and normal pregnancies (*n* = 6) during cesarean section. The samples were stored at −80°C for subsequent analysis. Participants with chronic hypertension, urinary tract infection, diabetes mellitus or other pregnancy complications were excluded from this study. Detailed baseline clinical characteristics of all participants are summarized in Table 1. The research was approved by the Ethics Committee of Sichuan Provincial Women’s and Children’s Hospital. Both verbal and written informed consent was obtained before the operation.

**Table 1.** Baseline clinical characteristics of study participants.

	PE	Blood pressuer	Proteinuria	Times of pregnancy	Age
1	Yes	109/71	Positive	G1P0	37
2	Yes	120/76	Positive	G1P0	37
3	Yes	149/98	Positive	G1P0	29
4	Yes	126/90	Positive	G1P0	27
5	Yes	144/89	Positive	G2P1	33
6	Yes	121/75	Positive	G3P1 + 1	39
7	No	119/62	Negative	G1P0	31
8	No	116/60	Negative	G2P0 + 1	25
9	No	110/62	Negative	G2P1	27
10	No	110/62	Negative	G3P3	24
11	No	125/71	Negative	G2P1	29
12	No	105/62	Negative	G2P1	28

## LncRNA microarray analysis

To profile lncRNA expression, total RNA was isolated from six pairs of preeclamptic and normal placental tissues using the mirVana™ RNA isolation kit (AM1561, Thermofisher). Subsequently, the total RNA was synthesized into cDNA and hybridized to microarray analysis according to the REPLI-g Single Cell Kit (qiagen 150,343). A list of lncRNAs was generated by microarray significance analysis and differentially expressed lncRNA was analyzed by random variance model.

## Cell culture

HUVEC were acquired from ATCC (Manassas, VA, USA) and grown in RPMI-1640 medium (E600028, Sangon Biotech) containing 10% FBS (E600052, Sangon Biotech) and 1% penicillin–streptomycin solution (E607011, Sangon Biotech). The cells were incubated at 37°C in a 5% CO_2_ humidified incubator.

## Cell transfection

Overexpression lentivirus/plasmids including oe-UNC5C-AS1, oe-EMP1, miR-148a-3p mimics, as well as small interfering RNAs sequence targeting UNC5C-AS1 (si-UNC5C-AS1) and their negative control were ordered from GenePharma Inc (Shanghai, China). Lipofectamine 3000 reagent (E607404, Sangon Biotech) was used for cell transfection according to the manufacturer’s protocol.

## Cell counting Kit-8 (CCK-8) assay

Cell viability was detected following the protocol of Cell Counting Kit-8 (CK04, Solarbio). HUVEC (4 × 10^3^ cells) were transferred into 96-well plates and cultured at 37°C for 24 h. After addition of 10 μL of CCK-8 solution, the cells were incubated for 2 h at 37°C. Absorbance was detected at 450 nm with a microplate autoreader (BioTek Instruments, USA).

## 5-ethynyl-2’-deoxyuridine (EdU) assay

HUVEC proliferation was evaluated according to the directions of SF594 EdU assay kit (CA1174-B, Solarbio). In brief, HUVEC (3 × 10^3^ cells/well) were transferred into 96-well plates and cultured for 24 h. Afterward, the cells were incubated in an EdU buffer solution for 2 h, and processed with 4% paraformaldehyde and 0.5% Triton X-100. After removing the medium, cells were stained with apollo staining solution for 30 min. DAPI buffer was applied for DNA staining. Images were analyzed under a fluorescence microscope (IX51, OLYMPUS).

## Cell cycle analysis

Cell cycle distribution was examined via cell cycle assay kit (40301ES60, yeasen). HUVEC were harvested and exposed to pre-cooled 70% ethanol for 24 h. Subsequently, the fixed cells were resuspended by 500 μL PBS and stained with 10 μL PI/RNase dye solution for 30 min at 37°C. Finally, cell cycle distribution was assayed by a flow cytometer (FACSCanto II, BD).

## Cell apoptosis analysis

Annexin V-FITC/PI staining kit (40302ES50; yeasen) was applied to assess cell apoptosis. Cells were resuspended in 300 μL binding bufferand double-stained with 5 μL Annexin V-FITC and 10 μL PI buffer, followed by flow cytometry analysis (FACSCanto II, BD Biosciences).

## Scratch assay

The scratch assay was utilized to evaluate the migratory ability of cells. Cells were transferred into 6 well plates and grown in RPMI-1640 medium until full monolayer formation. Subsequently, a 200 μL pipette tip was employed to scratch a line vertically across the plate. After washing it with PBS to eliminate detached cells, the plates were incubated for an additional 24 hours. Images of the wound were captured at 0 and 24 hours by a light microscope (IX51, OLYMPUS).

## Transwell assay

The invasive potential of HUVEC was evaluated by Transwell assay. After resuspension in serum-free RPMI-1640, cells were plated on Matrigel-precoated (50 μL) transwell chambers (Corning 3422). The lower chamber was filled with 500 μL of RPMI-1640 supplemented with 10% FBS. Following a 48-hour incubation at 37°C, the invading cells on the underside of the membrane were fixed with 4% paraformaldehyde and stained with 0.1% crystal violet dye (C0121, Beyotime) for 10 min. The dyeing cells were counted and representative images were obtained by inverted microscope (IX51, OLYMPUS).

## Tube formation assay

Tube formation was performed to evaluate the angiogenic capacity of HUVECs. Briefly, 24-well plates were precoated with Matrix-Gel™ (C0376, Beyotime) and polymerized at 37°C for 30 min. Next, HUVECs were resuspended in complete medium at a density of 1 × 10^5^ cells per well and seeded onto the polymerized gel. After 6 h of incubation at 37°C with 5% CO_2_, tubular structures were observed under an inverted microscope. Tube length was analyzed using ImageJ software

## Confocal fluorescence imaging

HUVEC were planted on the laser confocal culture dish (J40201, Jingan Biological). Following fixation with 4% paraformaldehyde for 15 min, samples were permeabilised using 0.1% Triton X-100 for 5 min and blocked with BSA solution for another 1 h at room temperature. Next, cells were washed with PBS and incubated with anti-tubulin antibodies (T3526; Sigma-Aldrich) at 4°C overnight. For cytoskeletal visualization, F-actin was labeled with Alexa Fluor® 488-phalloidin (ab150077, Abcam) for 15 min, while nuclei were stained with Hoechst 34,580 (63493, Sigma-Aldrich) and the cytoskeleton of HUVEC cells was observed by laser confocal fluorescence microscopy (K1-Fluo ABM, Nanoscope Systems).

## Dual-luciferase reporter assay

Bioinformatic analysis of potential interactions between miR-148a-3p and UNC5C-AS1/EMP1 was performed using StarBase. The wild-type UNC5C-AS1 (UNC5C-AS1-WT) or mutant UNC5C-AS1 (UNC5C-AS1-MUT) promoter sequences were cloned into a pmirGLO vector (Promega) and introduced into the 293T cells with miR-148a-3p mimics (or mimics-NC) using Lipofectamine 3000 Reagent (E607404, Sangon Biotech) for 48 h. Next, the luciferase activity was confirmed using Dual-Luciferase® Reporter Assay System (E1910, Promega, USA).

## Fluorescence in situ hybridization (FISH)

FISH assays were conducted by the lncRNA FISH Probe Mix (C10920, RiboBio). In brief, cells were fixed with 4% paraformaldehyde and permeabilized. Following 30 min of prehybridization at 37°C, the cells were treated with the fluorescently labeled probe overnight. The nuclei were restained with DAPI staining, and the localization of UNC5C-AS1 in placental tissues was observed by laser confocal microscopy (LSM 800 with Airyscan; Zeiss, Germany).

## PE mice model

Female ICR mice (10 weeks old) provided by Vital River Technology were used for the PE mouse model. All animals were housed in a room with controlled temperature (25 ± 2°C, 60 ± 5% humidity), with free access to water and food during a 12-hour light/dark cycle. During estrus, females and males were paired in a 2:1 ratio, and the day of vaginal pessary dislodgement was defined as GD0.5. Pregnant mice were subsequently divided into 4 groups: control, PE, PE+oe-NC, and PE+oe-UNC5C-AS1 groups (*N* = 5). Mice in the PE model group were injected subcutaneously for 10 days beginning at E8.5 with L-NAME (100 mg/kg, N5751, Sigma-Aldrich), and mice in control group were administered equivalent volumes of saline. Moreover, mice in the PE+oe-NC and PE+oe-UNC5C-AS1 groups were injected with lentivirus packaged plasmids pCDNA3.1-UNC5C-AS1 or pCDNA3.1-negative control (NC) through tail vein. Mice were euthanized by with 5% isoflurane for 5 min to collect the samples. Placenta or blood specimens were obtained for further analysis. All animal procedures were conducted by the Sichuan Provincial Women’s and Children’s Hospital Ethics Committee (20230807-211).

## Blood pressure measurement and urine analysis

Systolic blood pressure (SBP) and diastolic blood pressure (DBP) were determined via a tail-cuff volumetric tracer (Visitech BP2000 system, USA) at three time points: E5.5, E14.5 and E17.5. Urine on E17.5 at gestation was extracted for urine analysis referring to the instructions of Urine Protein Colourimetric Assay Kit (E-BC-K252-M, Elabscience®). The absorbance (OD) value at 595 nm was analyzed.

## Hematoxylin and eosin (H&E) staining

The placenta and kidney tissues were fixed in 4% paraformaldehyde, paraffin-embedded and cut into 2–3 μm thickness. Next, paraffin sections were treated with dewaxing, hydration, and stained with hematoxylin staining (C0105S, Beyotime) for 5 minutes. After staining, the sections were put into 1% hydrochloric acid alcohol solution for differentiation treatment, and stained with eosin solution for 2 minutes. Finally, neutral gum was used for sealing. Tissue sections were analyzed under a microscope (IX51, Olympus).

## Quantitative real-time PCR (qRT-PCR) analysis

Total RNA from placental tissues and HUVEC were extracted by Trizol (DP424, TIANGEN), while cDNA was synthesized using Super M-MuLV First Strand cDNA Synthesis Mix (B110023, Sangon Biotech). Then, real-time PCR was carried out using PerfectStart® Green qPCR SuperMix (AQ601-04, Transgen) with CG Real Time PCR (Heal Force). The relative mRNA levels (UNC5C-AS1, miR-148a-3p and EMP1) were analyzed by the 2^−∆∆Ct^ method. Primers are presented in Table 1.

## Western blot

Total protein extraction of placental tissues and HUVEC was conducted by RIPA Lysis Buffer (AS1004, ASPEN) and analyzed by Easy II Protein Quantitative Kit (BCA) (DQ111-01, TIANGEN). Equal quantities of proteins were separated on SDS-PAGE gels and transferred onto PVDF membranes (IPVH00010, Millipore). The membranes were subjected to blocking with 5% skimmed milk for 1 h, followed by overnight incubation at 4°C with primary antibodies against: VEGFA (AF5131, Affinity, 1:1000), MMP2 (AF5330, Affinity, 1:1000), Vimentin (AF7013, Affinity, 1:1000), EMP1 (ab202975, Abcam, 1:300) and GAPDH (10494–1-AP, Proteintech, 1:10000). Next, the membranes were treated with corresponding secondary antibodies. The proteins were captured using EasySee® Super Western Blot Kit (DW111-01, TIANGEN). Image J software was used to analyze band signals.

## Statistical analysis

Our data was shown as mean ± standard deviation (SD) from three independent replicates. For comparisons involving two groups, statistical significance was evaluated using the two-tailed Student’s t-test. Multi-group comparisons were analyzed by one-way ANOVA with Tukey’s post hoc test. All statistical analyses were conducted by GraphPad Prism 8.0 software (La Jolla, CA, USA). Statistical significance was defined as *p* < .05.

## Results

### UNC5C-AS1 was downregulated in PE tissues and localized in endothelial cells

**Figure 1.**
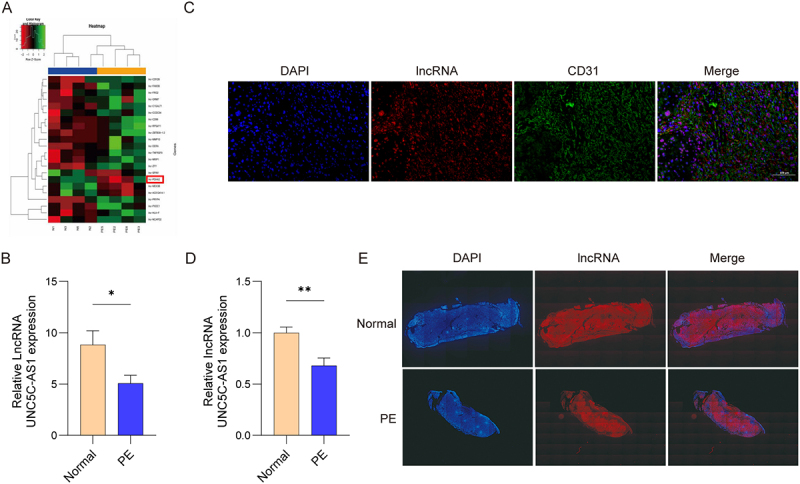
Expression of UNC5C-AS1 in PE tissues.

First, bioinformatic analysis was performed to analyze differentially expressed lncRNAs in tissues. We identified lnc PDHA2 (UNC5C-AS1) was remarkably down-regulated in PE tissues, relative to control group (Figure 1A). qRT-PCR was used to assess UNC5C-AS1 expression in 6 pairs of PE and paired normal placental tissues. As presented in Figure 1B, UNC5CAS1 expression showed significant reduction in PE tissues relative to normal controls. To further determine the distribution of UNC5C-AS1 in placental tissues, FISH analysis was performed. The findings revealed that UNC5C-AS1 co-localized with CD31 in endothelial cells (Figure 1C). Consistently, qRT-PCR and FISH further confirmed the low expression of UNC5C-AS1 in placental tissues of PE mice (Figure 1D,E). Our data demonstrate that UNC5C-AS1 contributes to the pathophysiological processes of PE.

(A) Heatmap revealing the differential expression of lncRNAs. (B) qRT-PCR analysis of UNC5C-AS1 expression in the placental tissues from normal pregnancies and PE patients. (C) The cellular location of UNC5C-AS1 and CD31 in placental tissues of PE mice was confirmed using FISH (*N* = 5). (D) Relative level of UNC5C-AS1 in placental tissues from PE mice and normal mice (*N* = 5). (E) UNC5C-AS1 expression was determined using FISH. *N* = 6, *p < .05, **p < .01.

### UNC5C-AS1 promoted HUVEC migration, invasion, and angiogenesis

To validate the biological functions of UNC5C-AS1 in PE, we overexpressed/knocked down UNC5C-AS1 expression by transfecting with oe-UNC5C-AS1 or si-UNC5C-AS1 into HUVEC. We observed that UNC5C-AS1 was overexpressed in oe-UNC5C-AS1 transfected HUVEC, while down-regulated by UNC5C-AS1 silencing (Figure 2A). The results from CCK-8 and EdU assay exhibited no significant effect on HUVEC viability and proliferation after overexpression or knockdown of UNC5C-AS1 (Figure 2B,C). In addition, we observed similar results in cell apoptosis and cell cycle in flow cytometry analysis (Figure 2D,E). We next examined the migratory and invasive abilities of HUVEC. The results showed that cell migration and invasion were remarkably enhanced by up-regulation of UNC5C-AS1 expression, compared to negative control. Conversely, the migration and invasion of HUVEC were obviously inhibited after UNC5C-AS1 silencing (Figure 2F,G). HUVEC capillary-like tube formation assay further confirmed that UNC5C-AS1 overexpression promoted HUVEC capillary-like tube formation, which was inhibited after UNC5C-AS1 silencing (Figure 2H). Confocal fluorescence assay showed that the cytoskeleton was disorganized, and there was a partial absence of microfilaments in si-UNC5C-AS1-transfected HUVEC (Figure 2I). Moreover, we determined the vascular permeability factors using Western blot, including VEGFA, MMP2, and Vimentin. As presented in Figure 2J, VEGFA, MMP2, and Vimentin exhibited an increased expression in oe-UNC5C-AS1 transfected HUVEC, while suppressed after UNC5C-AS1 silencing. Together, these data demonstrate that UNC5C-AS1 was involved in the PE development by regulating HUVEC migration, invasion and angiogenesis.

**Figure 2.**
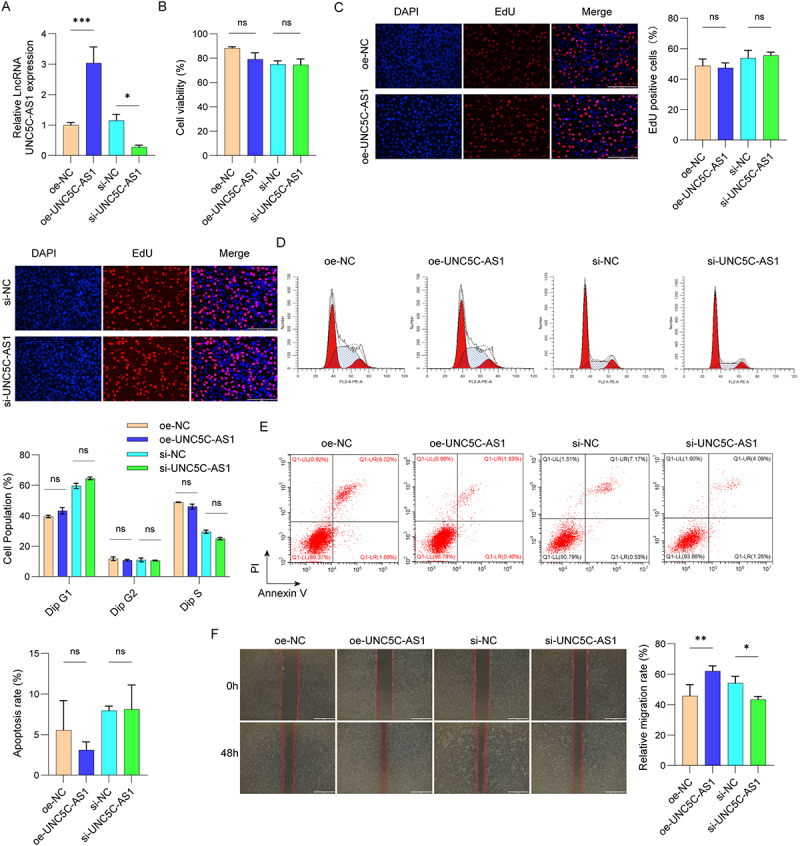
Effect of UNC5C-AS1 on HUVEC biological functions.

(A) HUVEC were transfected with oe-UNC5C-AS1 or si-UNC5C-AS1, and cell transfection efficiency was evaluated using qRT-PCR. (B) Cell viability of HUVEC was measured by CCK-8 assay. (C) Proliferation ability of HUVEC was evaluated by EdU staining. (D-E) Flow cytometric analysis showed cell cycle and apoptosis of HUVEC. (F-G) Cell invasion and migration ability were detected by wound healing and transwell assays. (H) HUVEC angiogenesis was determined by tube formation assay. (I) Cytoskeleton of HUVEC were visualized by fluorescence microscopy. (J) Western blot analysis of the VEGFA, MMP2, and Vimentin expression levels. *N* = 3. *p < .05, **p < .01, ***p < .001.

**Figure 2.**
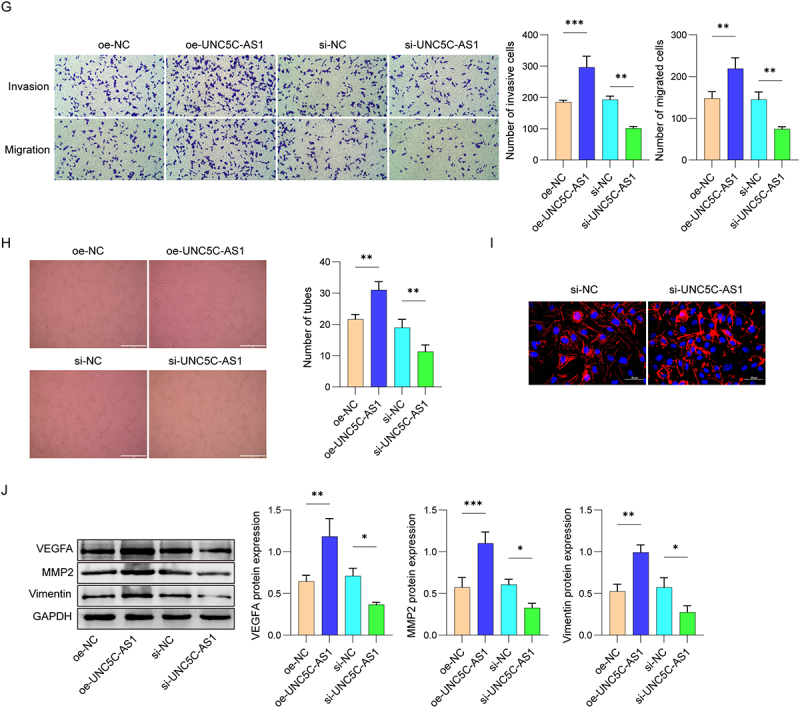
(Continued).

### UNC5C-AS1 regulated HUVEC biological functions via targeting miR-148a-3p

**Figure 3.**
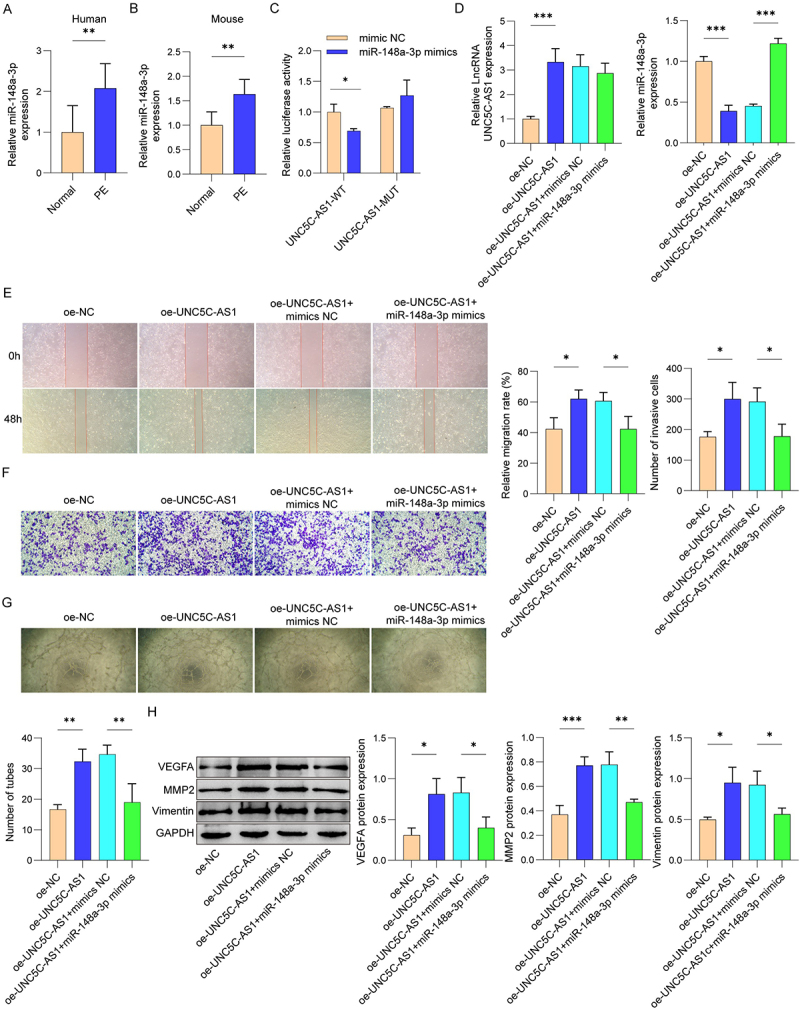
UNC5C-AS1 regulated HUVEC functions via its association with miR-148a-3p.

To illustrate the possible roles of UNC5C-AS1 in HUVEC functions, the downstream target gene of UNC5C-AS1 was verified through bioinformatic analysis. Our findings identified miR-148a-3p as a potential target of UNC5C-AS1. Notably, miR-148a-3p expression in the placental tissues from PE patients and PE mice was greatly enhanced, in contrast to the normal group (Figure 3A,B). Further dual-luciferase reporter assay displayed that miR-148a-3p mimics resulted in a great reduction in the luciferase activity in UNC5C-AS1-WT transfected 293T cells, but not in cells transfected with UNC5C-AS1-MUT, suggesting that miR-148a-3p directly targeted UNC5C-AS1 (Figure 3C). Subsequently, we detected the UNC5C-AS1 and miR-148a-3p levels in oe-UNC5C-AS1 and/or miR-148a-3p mimics transfected cells. UNC5C-AS1 overexpression enhanced its own level and decreased miR-148a-3p level, while miR-148a-3p mimics treatment had no effect on UNC5C-AS1 level, but elevated miR-148a-3p expression (Figure 3D). Then, the promotion of HUVEC migration and invasion by UNC5C-AS1 overexpression had been notably abrogated by miR-148a-3p mimics (Figure 3E,F). Besides, UNC5C-AS1 overexpression treatment obviously stimulated the HUVEC angiogenesis, which was suppressed by miR-148a-3p mimics transfection (Figure 3G). Also, up-regulation of VEGFA, MMP2, and Vimentin expression in oe-UNC5C-AS1 transfected HUVEC was partially reversed by miR-148a-3p mimics (Figure 3H). These data demonstrate that UNC5C-AS1 could regulate HUVEC migration, invasion, and angiogenesis via binding with miR-148a-3p.

(A-B) Relative RNA levels of miR-148a-3p in placental tissues from PE patients and PE mice were assessed via qRT-PCR. (C) The binding between UNC5C-AS1 and miR-148a-3p was predicted and confirmed by dual-luciferase reporter assay. (D) Relative expression of UNC5C-AS1 and miR-148a-3p in HUVEC transfected with oe-UNC5C-AS1 and/or miR-148a-3p mimics. (E-F) The migration and invasion of HUVEC were assessed using wound healing and transwell assays. (G) HUVEC angiogenesis was determined by tube formation assay. (H) Western blot for VEGFA, MMP2, and Vimentin expression. *N* = 3. *p < .05, **p < .01, ***p < .001.

### MiR-148a-3p targeted EMP1 to regulate HUVEC functions

Next, we illustrated the regulatory mechanism by which miR-148a-3p regulates HUVEC migration, invasion, and angiogenesis. Bioinformatic prediction suggested that EMP1 is a direct target of miR-148a-3p. EMP1 was downregulated in placental tissues from both PE patients and PE mice (Figure 4A,B). Figure 4C showed that miR-148a-3p mimic notably inhibited the relative luciferase activity in co-transfection of EMP1-WT and miR-148a-3p mimics, as opposed to the EMP1-WT and NC mimics group, while EMP1-mut did not change the luciferase activity in the presence of the miR-148a-3p mimics. Moreover, miR-148a-3p mimics elevated the expression of miR-148a-3p in HUVEC (Figure 4D). The expression of EMP1 was reduced in HUVEC after miR-148a-3p mimics transfection. However, this down-regulation was reversed after over-expression of EMP1 (Figure 4E). Further results suggested that HUVEC migration and invasive capacity were obviously inhibited after the transfection of miR-148a-3p mimics (Figure 4F,G). Meanwhile, the angiogenesis of HUVEC was inhibited after miR-148a-3p mimics treatment (Figure 4H). On the contrary, the migration, invasion, and angiogenesis of HUVEC were partly reversed after transfected with oe-EMP1. Besides, miR-148a-3p mimics markedly reduced the protein expression of VEGFA, MMP2 and Vimentin in HUVEC, which were partially rescued after EMP1 overexpression (Figure 4I). The observations demonstrate that miR-148a-3p regulated the migration, invasion, and angiogenesis of HUVEC by targeting EMP1.

**Figure 4.**
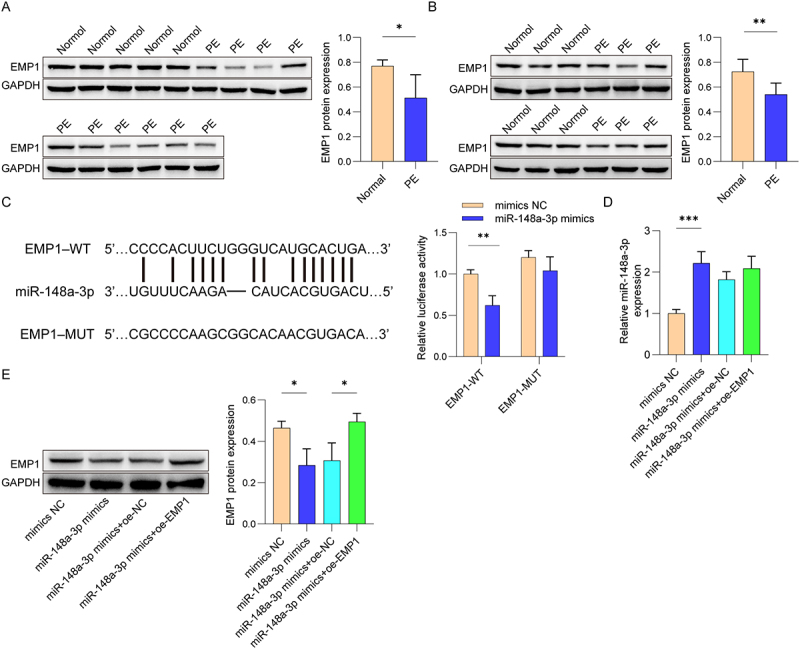
MiR-148a-3p promoted HUVEC migration, invasion and angiogenesis by targeting EMP1.

(A) Expression of EMP1 in placental tissues of PE patients and healthy individuals was determined using Western blot. (B) Expression of EMP1 in placental tissues of mice was determined using Western blot. (C) The binding sites between miR-148a-3p and EMP1 were predicted by starBase and confirmed by dual-luciferase reporter assay. (D) The transcription level of miR-148a-3p was measured by qRT-PCR. (E) Western Blot was used to evaluate the expression of EMP1 proteins. Cell migration (F) and invasion (G) were assessed by wound healing and transwell assay. (H) HUVEC angiogenesis was determined by tube formation assay. (I) The protein levels of VEGFA, MMP2, and Vimentin were measured by Western blot. *N* = 3. *p < .05, **p < .01, ***p < .001.

**Figure 4.**
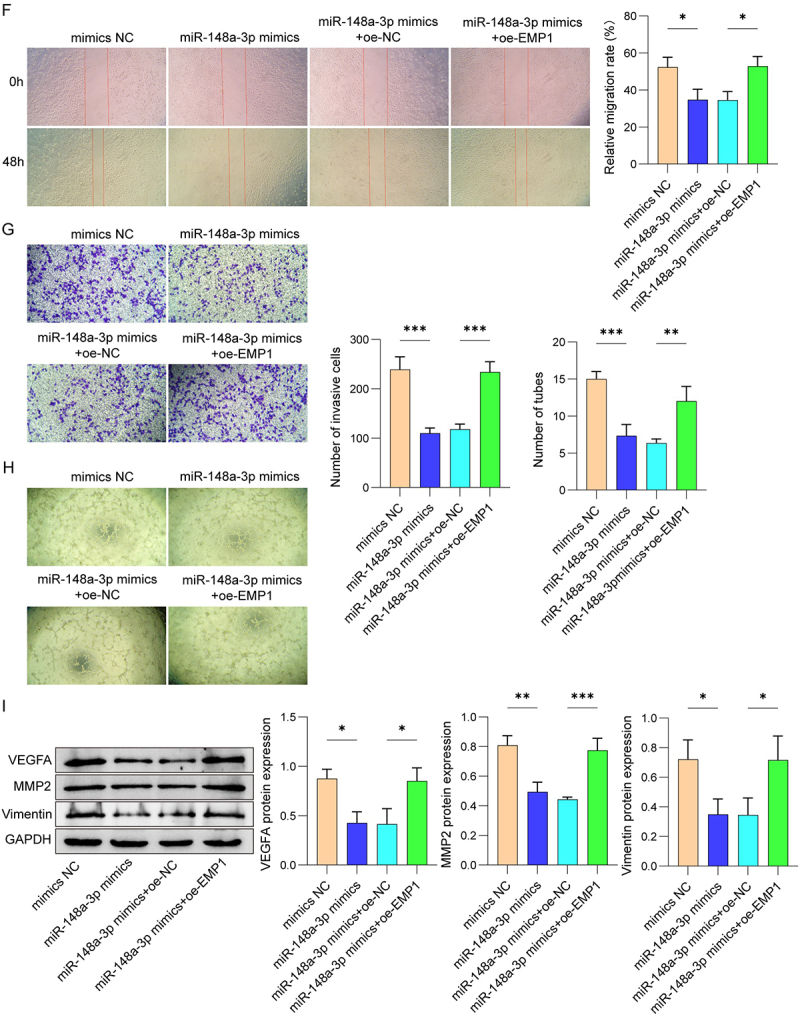
(Continued).

### UNC5C-AS1 regulated HUVEC function via miR-148a-3p/EMP1 axis

**Figure 5.**
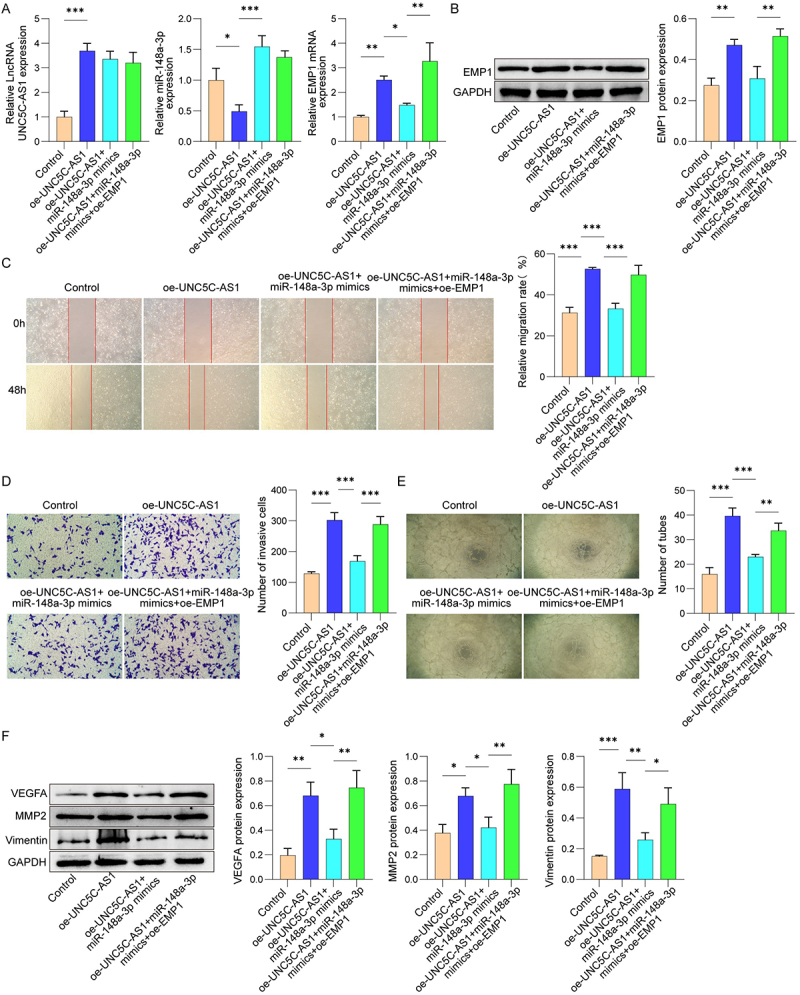
UNC5C-AS1 regulated HUVEC migration, invasion and angiogenesis by targeting miR-148a-3p/EMP1 axis.

To confirm whether UNC5C-AS1 regulates HUVEC migration, invasion, and angiogenesis via the miR-148a-3p/EMP1 axis, we conducted transfection with oe-UNC5C-AS1, miR-148a-3p mimics, or oe-EMP1. As displayed in Figure 5A, the UNC5C-AS1 and EMP1 level was increased, while miR-148a-3p level was suppressed upon UNC5C-AS1 overexpression. By contrast, miR-148a-3p mimics elevated miR-148a-3p levels and reduced EMP1 expression without altering UNC5C-AS1. Overexpression of EMP1 increased EMP1 expression but did not affect UNC5C-AS1 and miR-148a-3p levels. Furthermore, overexpression of UNC5C-AS1 enhanced the protein level of EMP1, which was reduced after transfection of miR-148a-3p mimics. Nevertheless, this suppression was partly abolished by EMP1 overexpression (Figure 5B). Next, we also evaluated the roles of UNC5C-AS1/miR-148a-3p/EMP1 axis on HUVEC biological behaviors. Results in Figure 5C-E suggested that overexpression of UNC5C-AS1 promoted migration, invasion, and tube formation ability of HUVEC, which were inhibited by miR-148a-3p mimics transfection. Nevertheless, treatment with oe-EMP1 remarkably restored the miR-148a-3p mimics-mediated suppression on HUVEC biological behaviors (Figure 5C-E). UNC5C-AS1 overexpression promoted VEGFA, MMP2 and Vimentin expression, an effect that was inhibited by miR-148a-3p mimics. Notably, EMP1 overexpression partially rescued the expression of these proteins (Figure 5F). These findings indicate that UNC5C-AS1 targeted miR-148a-3p/EMP1 to regulate the migration and invasion of HUVEC.

(A) qRT-PCR was applied to measure the mRNA levels of UNC5C-AS1, miR-148a-3p and EMP1. (B) EMP1 expression in HUVEC was determined by using Western blot. (C) The wound healing assay was performed to detect the migration ability of HUVEC. (D) Transwell assay was used to detect the invasive ability of HUVEC. (E) HUVEC angiogenesis was determined by tube formation assay. (F) The protein levels of VEGFA, MMP2, and Vimentin were measured by Western blot. *N* = 3. *p < .05, **p < .01, ***p < .001.

### UNC5C-AS1 alleviated injury in mice by targeting miR-148a-3p/EMP1 axis

**Figure 6.**
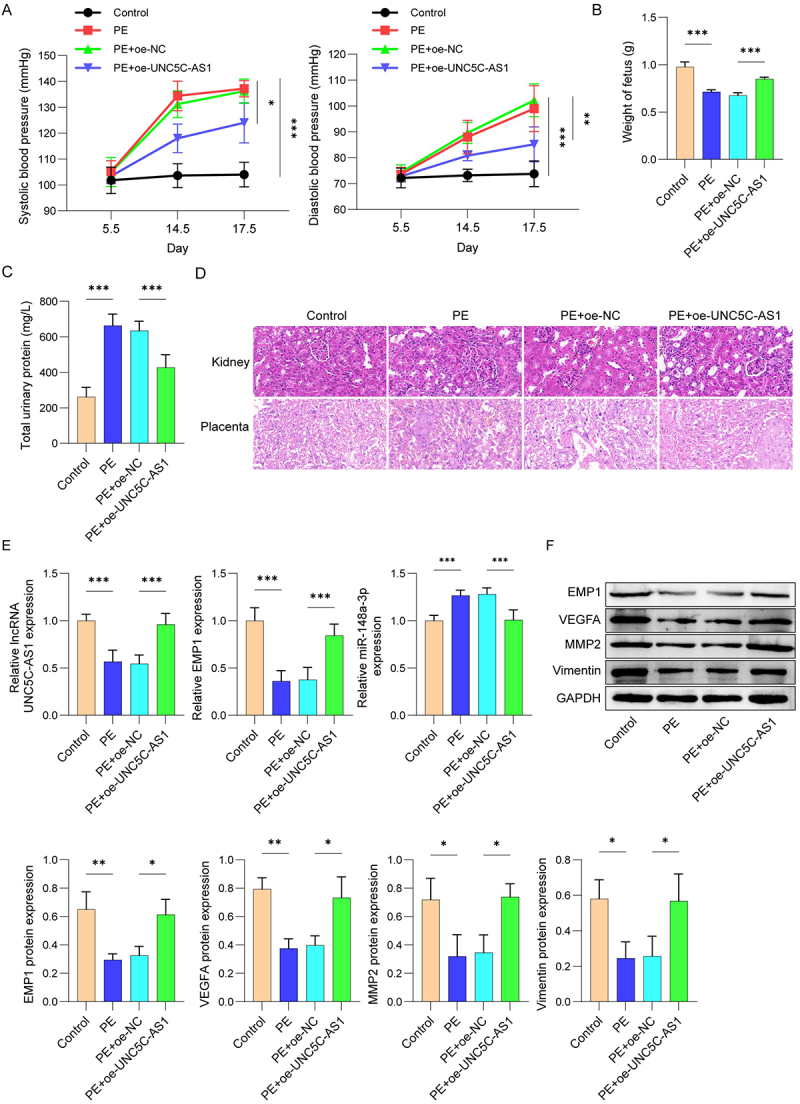
UNC5C-AS1 overexpression alleviated PE progression in PE mice by regulating miR-148a-3p/EMP1 axis.

To further explain the therapeutic effects of UNC5C-AS1 overexpression on PE, we established a PE mouse model. UNC5C-AS1 plasmid was treated into the PE mice. SBP and DBP of mice at E5.5, E14.5 and E17.5 were detected by tail cuff plethysmography. Our data exhibited increased SBP and DBP in PE group compared with the control mice but were partially normalized after UNC5C-AS1 transfection (Figure 6A). Addition, the weight of fetus and total urinary protein in PE mice were assessed. These measurements revealed decreased weight of fetus and increased total urinary protein levels in PE mice relative to the control mice, both of which were partially rescued by UNC5C-AS1 overexpression (Figure 6B,C). Histological analysis of placental and kidney tissues by H&E staining showed that PE mice exhibited placental infarction, necrotic area, and narrow blood blisters. In renal tissues, PE mice displayed glomerular constriction and narrowing of glomerular cystic cavity, compared with the control groups. However, we observed the inverse results in UNC5C-AS1 overexpression group (Figure 6D). UNC5C-AS1 and EMP1 were weakened while miR-148a-3p was elevated in the PE mice relative to the control mice (Figure 6E). In addition, consistent with expression in HUVEC, PE mice placentas showed downregulated VEGFA, MMP2, and Vimentin expression, in contrast to the control group, as determined by Western blot (Figure 6F). Nevertheless, these results were partially reversed after transfection of UNC5C-AS1. Our findings above indicate that UNC5C-AS1 overexpression may alleviate PE tissue injury by regulating miR-148a-3p/EMP1 axis.

The PE mice were injected with lentivirus overexpressing UNC5C-AS1, and the empty lentiviral vector as a control. (A) SBP and DBP were determined by the tail-cuff technique. (B-C) Weight of fetus and total urinary protein of pregnant rats were measured. (D) Placenta tissues and kidney tissues were observed by H&E staining. (E) The expression levels of UNC5C-AS1, miR-148a-3p and EMP1 were assessed by qRT-PCR. (F) Protein expressions of VEGFA, MMP2, and Vimentin in placentas were analyzed by Western blot. *N* = 5. *p < .05, **p < .01, ***p < .001.

## Discussion

PE is a complex gestational disorder caused by a variety of factors that leading to cardiovascular disease and organ damage, ultimately leading to termination of pregnancy [19]. However, the etiology of PE has not been fully elucidated. Increasing evidence suggests that lncRNAs are implicated in the progression of PE [20]. Our research is the first to identify UNC5C-AS1 as a regulatory factor in PE, suggesting that UNC5C-AS1 regulated HUVEC function through miR-148a-3p/EMP1 axis. Our study provided new perspectives on understanding PE progression.

Various lncRNAs have been reported to be associated to the pathogenesis of PE, including APOA1-AS [21], TARID and DUXAP8 [22,23]. APOA1-AS and lncRNA DUXAP8 were up-regulated in the placental tissue of PE. DUXAP8 overexpression inhibited trophoblast proliferation, migration, and invasion in HTR-8/SVneo and JAR cells. In this study, UNC5C-AS1 expression was markedly decreased in PE tissues relative to normal pregnancies placenta tissues. To the best of our knowledge, this is the first report elucidating the functional role of UNC5C-AS1 in PE. The function of lncRNAs is closely linked to their subcellular localization. Our data revealed that UNC5C-AS1 localizes to both the cytoplasm and nucleus, suggesting its potential involvement in ceRNA regulatory networks. Furthermore, FISH confirmed the co-localization of UNC5C-AS1 with the endothelial marker CD31, highlighting its specific presence in vascular endothelial cells. UNC5C-AS1Endothelial cells play crucial roles in placental vascular function [24], and endothelial dysfunction is considered a central pathological feature of PE, contributing to its characteristic symptoms of hypertension and proteinuria [25,26]. Therefore, improving endothelial cell function is a promising therapeutic strategy for PE. Liu et al revealed that inhibition of PPP2R2A regulates the proliferation, apoptosis, and angiogenesis of mesenchymal stem cells in PE [27]. Moreover, Huang et al have found that sFlt-1-Exo significantly inhibited HUVEC migration and tube formation, as well as triggered a PE-like phenotype [28]. Our findings were similar to these results, revealing that UNC5C-AS1 overexpression enhanced HUVEC migratory and invasive capacities, whereas UNC5C-AS1 silencing exhibited the opposite effect. In addition, our study further revealed that knockdown of UNC5C-AS1 inhibited angiogenesis and decreased the expression levels of vascular permeability factors in HUVEC. To explain the anti-PE effect of UNC5C-AS1 in vivo, ICR mice were subjected to subcutaneous injection of L-NAME to establish a PE model. We found that UNC5C-AS1 expression was reduced in placental tissues of PE mice. Meanwhile, UNC5C-AS1 treatment reduced SBP, DBP, fetal mouse weight, and urinary protein levels of PE rats, accompanied by an improved PE phenotype and attenuated tissue damage in PE mice, which is similar to the main clinical features observed in PE patients and other researches [29,30]. These findings revealed the essential regulatory role UNC5C-AS1 in the pathological of PE.

Recent studies have increasingly demonstrated the main pathogenic roles of lncRNAs are regulated through their interference with gene expression by targeting complementary miRNAs [31]. Wei et al. demonstrated that lncRNA PVT1 promoted the placental trophoblasts growth via miRNA-24-3p/HSD11B2 axis [32]. Report by Zhang et al. showed that over-expression of lncRNA NORAD enhanced trophoblast proliferation and invasive capacity via miR-202-5p/FXR1 axis [33]. In line with these findings, we identified miR-148a-3p as a direct target of UNC5C-AS1 through integrated bioinformatic prediction and experimental validation. MiR-148a-3p dysregulation has been reported in various cancers, including gastric cancer [34], non-small-cell lung cancer and bladder cancer [35,36]. Besides, miR-148a-3p was involved in endothelial dysfunction in human placental tissues and targets functional genes, such as SIRT7 and ROCK1 [36,37]. Our study further substantiates its role in PE, demonstrating that miR-148a-3p was significantly upregulated in PE tissues from both humans and mice. MiR-148a-3p expression levels in endothelial cells were reduced after UNC5C-AS1 overexpression. Notably, miR-148a-3p mimics significantly abrogated the regulatory effects of UNC5C-AS1 overexpression on endothelial cell migration, invasion, angiogenic capacity, and vascular permeability factors protein expression. These findings collectively delineate a novel UNC5C-AS1/miR-148a-3p regulatory axis in PE pathogenesis. Selvakumar et al. also illustrated the role of microRNA-based therapeutics in the treatment of preeclampsia [38]. Our study highlights its translational relevance for small RNA therapeutics in PE. Advances in delivery platforms – such as exosome or nanoparticle-based systems – may further enhance the feasibility and specificity of such interventions in a clinical setting.

It is well known that miRNA binds with target genes to regulate functions. Thus, the precise roles of miR-148a-3p in mediating HUVEC behavior may be attributed to different targeted genes. Bioinformatic analysis identified EMP1 as a miR-148a-3p target. EMP1 is an integral membrane glycoprotein that exerts crucial functions in human tumors. Liu et al have found that EMP1 promotes the ovarian cancer cells proliferation and invasion via the MAPK pathway [39]. Results in Liu et al also revealed that circ100284 enhances osteosarcoma cells invasion and migration via knock-downing EMP1 expression [40]. Here, we found that EMP1 is downregulated in placental tissues from both PE patients and mice. The results of rescue experiments indicated that EMP1 overexpression reversed the effects of miR-148a-3p mimics on HUVEC migration, invasion of angiogenesis. These findings above suggested that UNC5C-AS1 contributes to the progression of PE via regulating endothelial cell migration invasion through competitive binding of miR-148a-3p/EMP1. However, the involvement of miR-148a-3 and EMP1 in the regulatory mechanism of PE needs to be further explored.

In conclusion, our study demonstrates that UNC5C-AS1 regulates HUVEC migration, invasion, and angiogenesis via the miR-148a-3p/EMP1 axis, highlighting its potential as a therapeutic target for PE. However, several limitations should be acknowledged. First, the clinical sample size was relatively small, which may affect the generalizability of the findings. Future studies with larger cohorts are needed to validate these results. Second, while HUVECs are a widely used model, primary trophoblasts or maternal-fetal interface endothelial cells may better reflect PE pathophysiology. Third, the L-NAME-induced PE model, though widely used, does not fully recapitulate the complexity of human PE. Fourth, the downstream signaling pathways of EMP1, such as PI3K/AKT or MAPK, remain to be elucidated. Finally, whether UNC5C-AS1 regulates PE through additional mechanisms requires further exploration. Despite these limitations, our findings provide a foundation for future research and suggest that UNC5C-AS1-based interventions may hold promise for the treatment of PE.

## Data Availability

The datasets used or analyzed during the current study are available from the corresponding author on reasonable request.
